# Efficacy of low-dose local clindamycin in different times for microbial decontamination of autogenous particulate bone graft

**DOI:** 10.1186/s40729-020-00263-1

**Published:** 2020-10-30

**Authors:** Hassan Mohajerani, Gholamreza Irajian, Fatemeh Latifi, Faramarz Masjedian, Reza Tabrizi

**Affiliations:** 1 Department of Oral and Maxillofacial Surgery, Shahid Beheshti University of Medical Sciences, Velenjak, Tehran, Iran; 2 Department of Microbiology, Iran University of Medical Sciences, Tehran, Iran

**Keywords:** Decontamination, Bone, Graft, Clindamycin, Microorganism

## Abstract

**Background:**

Clindamycin in low concentration (20 μg/mL) is safe for vitality and osteogenic potential of bone cells. The aim of this study was to evaluate the efficacy of local clindamycin (20 μg/mL) in two different exposure times, for microbial decontamination of particulate bone graft, collected during implant site preparation.

This non-randomized parallel-group study was conducted on samples from 17 patients. The particulate bone collected during implant site preparation was divided into three portions by weight: in group S1, the particulate bone was immersed in thioglycolate broth without any antibiotic treatment; in group S2, the collected particulate bone was irrigated with 100 mL clindamycin solution (20 μg/mL); and in group S3, the collected particulate bone was soaked in one ml clindamycin solution (20 μg/mL) for 3 min. Samples in the three groups were cultured in aerobic and anaerobic media and species and CFU count of isolated bacteria were determined.

**Results:**

Analysis of the data demonstrated a significant difference among the three groups in the mean count of total microorganisms (*P* = 0.001). The difference in the mean count of anaerobic and aerobic microorganisms in the three groups was statistically significant as well (*P* = 0.001). *Pseudomonas aeruginosa* was the only microorganism that was not affected with the mentioned antibiotic.

**Conclusions:**

Local use of low-dose clindamycin (20 μg/mL)—irrigation or 3 min immersing—is effective for the decontamination of particulate bone grafts.

## Background

Nowadays, reconstruction of bone defects in oral surgery is one of the prevalent procedures, and according to the literature, using natural bone is superior to the commercial materials. Donor site defect is the most important disadvantage of autogenous bone grafting. Bone chips, derived from drilling, can serve as a suitable source for grafting without donor site morbidity [1]. They can be collected during dental implant surgery using a bone collector [2] or from the drill threads. The collected particulate bone can be used as an autogenous bone graft—alone or in combination with commercial materials—to cover the defects around implants. However, the risk of microbial contamination of the collected bone is a matter of concern. The oral cavity averagely contains 10^11^ bacteria in each gram of the plaque and 10^8^ bacteria in each milliliter of the saliva. Despite applying all the principles, contamination of bone grafts—either collected as bone particles or harvested as a whole piece—with the normal bacterial flora occurs [3].

Several techniques have been suggested to reduce the risk of bone contamination such as preoperative rinse of the oral cavity with chlorhexidine mouthwash [4], antibiotic prophylaxis [5, 6], and a stringent aspiration protocol [7].

As there is a transient decrease in blood supply to the surgical site, serum concentration of oral antibiotics is not sufficient for antimicrobial effect on the reconstructed regions; therefore, local administration of antimicrobial agents was considered [8].

Due to the disadvantages of local betadine and chlorhexidine on wound healing, cell vitality, and osteogenic potential, using antibiotic solutions as a decontamination agent for bone grafts is wise. Selecting the correct drug in an accurate concentration and precise contact time—harmless to the cell vitality and their osteogenic potential—is important.

Clindamycin is commonly used as a prophylactic antibiotic in maxillofacial surgery. It has excellent bone and soft-tissue penetration after single-dose administration [9].

Clindamycin in low concentration not only has no negative effect on cellular proliferation and metabolism, but also induces cell differentiation [10].

According to the in vitro examinations on primary human osteoblasts (PHO) and cell lines after exposure to different antibiotics, and due to the minimum inhibitory concentration (MIC) of clindamycin, a solution consisting 20 μg/mL of clindamycin was recommended for local decontamination of the bone grafts [11].

Therefore, the aim of this study was to evaluate the efficacy of local clindamycin—qualitatively and quantitatively—for decontamination of collected particulate bone grafts. Two different exposure times were selected and colony-forming units (CFU) counts of isolated spices after each exposure to the mentioned antibiotic were determined.

## Methods

The authors designed a non-randomized parallel-group study. The samples were derived from the population of patients presenting to the Oral and Maxillofacial Surgery Department of Shahid Beheshti Univercity of Medical Science (Tehran, Iran), between September 1, 2018, and November 30, 2018. The research protocol was approved by the Committee of Medical Ethics Group of dentistry school. Patients eligible for study inclusion had an edentulous area at the first molar site of the mandible and were candidates for dental implant placement. Patients were excluded from the study if they had periodontitis, poor oral hygiene, history of previous bone augmentation at the implant site, or systemic diseases affecting the immune system or bone metabolism. All surgical procedures were performed by the same oral and maxillofacial surgeon.

### Surgical approach

Patients received 2 g amoxicillin (Dana Co., Tabriz, Iran) orally 1 h before surgery. All patients used 10 mL of 0.2% chlorhexidine gluconate mouth rinse (Kemphor CHX, Pinseque, Spain) for 2 min. Local anesthesia (2% lidocaine with 1/80,000 epinephrine) was administered. A crestal incision was made on the edentulous alveolar ridge at the first molar area of the mandible.

During the preparation of the implant site with standard surgical drills (Straumann, Switzerland), 0.9% saline solution was used for irrigation and cooling of drills and preparation site.

According to stringent aspiration protocol [7], a sterile metal suction tip was used continuously to suction saliva and tissue fluids and reduce the risk of contamination of collected particulate bone with the oral microbial flora.

The particulate bone of each patient, collected from treads of twist drills, was divided into three equal portions by weight. Each portion was treated with clindamycin solution (20 μg/mL) in a specific time (group S1, 2, 3). The first sample only contained the collected particulate bone without any treatment in thioglycolate broth (group S1) as a control group. In the second group (group S2), the collected particulate bone was irrigated with 100 mL of clindamycin solution (20 μg/mL) and was then immersed in thioglycolate broth. In the third group (group S3), the collected particulate bone was soaked in 1 mL clindamycin solution (20 μg/mL) for 3 min and then was immersed in thioglycolate broth. All samples were placed in sterile boxes and transferred to a laboratory for microbial analysis. Thus, each patient had three samples for microbial evaluation.

### Laboratory procedure

All test tubes contained thioglycolate broth and were boiled before the placement of samples to remove oxygen from the tubes for the culture of anaerobic microorganisms. All samples were vortexed for 30 s to homogenize solutions and were then serially diluted (1:5, 1:10, 1:100, and 1:1000). To culture aerobic and anaerobic microorganisms, half of the solution was cultured on MacConkey agar and the other half on blood agar. For anaerobic culture, samples were retained in an anaerobic jar (85% nitrogen and 15% CO2) for 24–48 h at 35–37 °C. After culture, microorganisms were identified based on their biochemical characteristics and sensitivity to discs.

### Statistical analysis

Statistical analyses were performed using SPSS version 19 (SPSS Inc., IL, USA). Nonparametric tests were applied because the data were not normally distributed. Non-parametric Friedman test was used to compare microbial count among the three groups. Pairwise comparisons were made using the Wilcoxon signed-rank test and *P* value adjustment was done by the Bonferroni method.

## Results

Particulate bone samples were derived from seventeen patients (13 males and four females), and the mean age was 48.35 with SD16.8 years. Evaluation of total samples in the three groups revealed 27 microbial species consisting of 17 aerobic species, nine anaerobic species, and one type of fungus (Table 1).

**Table 1:** Various microorganisms isolated from the collected bone after culture

Microorganisms	Aerobic species	Anaerobic species	***N***
**Gram-positive**	*Staphylococcus epidermidis* *Corynebacterium* spp. *Streptococcus pneumoniae* *Staphylococcus capitis* *Micrococcus* spp. *Lactobacillus* spp. *Streptococcus* spp. *Staphylococcus saccharolyticus* *Enterococcus faecalis* *Staphylococcus saprophyticus* *Bacillus* spp. *Streptococcus oralis* *Lactococcus* spp.	*Propionibacterium propionicum* *Peptostreptococcus asaccharolyticus* *Propionibacterium* spp. *Peptostreptococcus* spp. *Actinomyces* spp. *Clostridium* spp.	19
**Gram-negative**	*Escherichia coli* *Neisseria* spp. *Klebsiella pneumoniae* *Pseudomonas aeruginosa*	*Fusobacterium* spp. *Veillonella* spp. *Bacteroides* spp.	7
**Total number**	17	9	26

Regardless of the time factor, all the microbial species population reduced after decontamination with clindamycin solution (20 μg/mL), except *Pseudomonas aeruginosa* (Table 2). Comparison of microorganisms CFU revealed a statistically significant reduction in nine microbial species between three groups.

**Table 2:** Comparison of the mean colony forming units of total microorganisms among the three groups

Microorganisms	Group S1	Group S2	Group S3	Friedman test
*Staphylococcus epidermidis*	0.94 ± 1.749	0.65 ± 1.222	0.12 ± 0.485	*P* = 0.018
*Corynebacterium* spp.	10.06 ± 8.050	4.06 ± 5.080	0.00 ± 0.00	*P* = 0.001
*Streptococcus pneumoniae*	0.76 ± 2.223	0.00 ± 0.00	0.00 ± 0.00	*P* = 0.135
*Escherichia coli*	0.29 ± 0.849	0.29 ± 0.849	0.18 ± 0.529	*P* = 0.368
*Staphylococcus capitis*	0.18 ± 0.728	0.18 ± 0.728	0.12 ± 0.485	*P* = 0.368
*Neisseria* spp.	4.00 ± 4.444	1.00 ± 1.904	0.00 ± 0.00	*P* = 0.001
*Micrococcus* spp.	1.65 ± 2.149	0.47 ± 1.328	0.00 ± 0.00	*P* = 0.001
*Klebsiella pneumoniae*	1.00 ± 1.803	0.76 ± 1.348	0.56 ± 1.176	*P* = 0.018
*Lactobacillus* spp.	1.12 ± 2.288	0.35 ± 1.455	0.00 ± 0.00	*P* = 0.023
*Streptococcus* spp.	0.41 ± 1.176	0.29 ± 0.849	0.29 ± 0.849	*P* = 0.135
*Staphylococcus saccharolyticus*	0.41 ± 1.176	0.18 ± 0.728	0.00 ± 0.00	*P* = 0.156
*Enterococcus faecalis*	0.41 ± 1.176	0.35 ± 0.996	0.35 ± 0.996	*P* = 0.368
*Staphylococcus saprophyticus*	0.65 ± 1.455	0.59 ± 1.326	0.06 ± 0.243	*P* = 0.061
*Pseudomonas aeroginosa*	0.18 ± 0.728	0.18 ± 0.728	0.18 ± 0.728	*P* = 1.0
*Bacillus* spp.	0.53 ± 1.58	0.12 ± 0.485	0.00 ± 0.00	*P* = 0.156
*Streptococcus oralis*	0.59 ± 2.425	0.00 ± 0.00	0.00 ± 0.00	*P* = 0.368
*Lactococcus* spp.	0.176 ± 0.727	0.00 ± 0.00	0.00 ± 0.00	*P* = 0.368
*Propionibacterium propionicum*	0.18 ± 0.529	0.00 ± 0.00	0.00 ± 0.00	*P* = 0.135
*Peptostreptococcus asaccharolyticus*	0.12 ± 0.485	0.00 ± 0.00	0.00 ± 0.00	*P* = 0.368
*Propionibacterium* spp.	2.058 ± 2.105	0.411 ± 0.939	0.00 ± 0.00	*P* = 0.001
*Peptostreptococcus* spp.	0.35 ± 0.996	0.29 ± 0.849	0.00 ± 0.00	*P* = 0.156
*Fusobacterium* spp.	0.41 ± 1.176	0.00 ± 0.00	0.00 ± 0.00	*P* = 0.135
*Veillonella* spp.	0.24 ± 0.664	0.12 ± 0.332	0.06 ± 0.243	*P* = 0.156
*Actinomyces* spp.	0.88 ± 1.536	0.06 ± 0.243	0.00 ± 0.00	*P* = 0.009
*Bacteroides* spp.	0.35 ± 1.057	0.00 ± 0.00	0.00 ± 0.00	*P* = 0.135
*Clostridium* spp.	0.12 ± 0.485	0.00 ± 0.00	0.00 ± 0.00	*P* = 0.368
*Candida*	0.35 ± 0.862	0.29 ± 0.849	0.12 ± 0.485	*P* = 0.097

Pairwise comparison of these nine species demonstrated that the only microorganism that has not been significantly reduced after irrigation with clindamycin solution (20 μg/mL) is *Klebsiella pneumoniae*, and immersion in 3 min was more effective.

In addition, CFU’s comparison of these nine species between S2 and S3 groups confirmed statistically significant reduction after 3 min contact, only in *Staphylococcus epidermidis* and *Corynebacterium* spp. population. It means that short time contact with clindamycin solution (20 μg/mL) could be effective for the most detected microorganisms.

The mean colony-forming units of total microorganisms was 26.76 with SD10.40(103) in the control group (S1), 10.64 with SD6.24(103) in the irrigation group (S2), and 1.94 with SD1.47(103) in the soaking group (S3). Analysis of the data revealed a significant difference among the three groups (*P* = 0.001) (Table 3).

**Table 3:** Comparison of the mean colony forming units of total microorganisms among the three groups

Microorganisms	Group S1	Group S2	Group S3	Friedman test
**Total CFU**	26.76 ± 10.40(10^3^)	10.64 ± 6.24(10^3^)	1.94 ± 1.47(10^3^)	*P* = 0.001

The mean colony-forming units of aerobic microorganisms was 23.53 with SD7.80(10^3^) in the control group (S1), 9.76 with SD5.71(10^3^) in the irrigation group (S2), and 2.00 with SD1.37(10^3^) in the soaking group (S3). Analysis of the data revealed a significant difference among the three groups in this respect (*P* = 0.001).

The mean colony-forming units of anaerobic microorganisms was 6.65 with SD 4.81(10^3^) in control group (S1), 1.29 with SD1.87(10^3^) in irrigation group (S2) and 0.06 with SD0.24(10^3^) in soaking group (S3). There was a significant difference among the three groups in this respect (*P* = 0.001, Table 4).

**Table 4:** Comparison of the mean colony forming units of aerobic and anaerobic microorganisms among the three groups

Microorganisms	Group S1	Group S2	Group S3	Friedman test
**Aerobic**	23.53 ± 7.80(10^3^)	9.76 ± 5.71(10^3^)	2.00 ± 1.37(10^3^)	*P* = 0.001
**Anaerobic**	6.65 ± 4.81(10^3^)	1.29 ± 1.87(10^3^)	0.06 ± 0.24(10^3^)	*P* = 0.001

The mean colony-forming units of Gram-positive microorganisms was 19.9 with SD 9.8(10^3^) in the control group (S1), 8.00 with SD 6.20(10^3^) in the irrigation group (S2), and 0.82 with SD 1.33(10^3^) in the soaking group (S3). There was a significant difference among the three groups respectively (*P* = 0.001).

The mean colony-forming units of gram negative microorganisms was 6.647 with SD 4.877(10^3^) in the control group (S1), 2.35 with SD 2.52(10^3^) in the irrigation group (S2), and 1.00 with SD 1.36(10^3^) in the soaking group (S3). There was a significant difference among the three groups in this respect too (*P* = 0.001, Table 5).

**Table 5:** Comparison of the mean colony-forming units of Gram-positive and Gram-negative microorganisms among the three groups

Microorganisms	Group S1	Group S2	Group S3	Friedman test
**Gram-positive**	19.94 ± 9.87(10^3^)	8.00 ± 6.20(10^3^)	0.82 ± 1.33(10^3^)	*P* = 0.001
**Gram-negative**	6.47 ± 4.77(10^3^)	2.35 ± 2.52(10^3^)	1.00 ± 1.36(10^3^)	*P* = 0.001

Pairwise comparisons of the groups for the mean colony-forming units of aerobic-anaerobic microorganisms and Gram-positive–Gram-negative microorganisms revealed significant differences between them (*P* = 0.001, Tables 6 and 7).

**Table 6:** Pairwise comparison of the mean colony-forming units of aerobic and anaerobic microorganisms between the groups

Groups	Aerobic microorganisms	Anaerobic microorganisms	Wilcoxon test
**Group S1**	23.53 ± 7.80(10^3^)	6.65 ± 4.81(10^3^)	*P* = 0.001
**S2**	9.76 ± 5.71(10^3^)	1.29 ± 1.87(10^3^)	
**Group S1**	23.53 ± 7.80(10^3^)	6.65 ± 4.81(10^3^)	*P* = 0.001
**S3**	2.00 ± 1.37(10^3^)	0.06 ± 0.24(10^3^)	
**Group S2**	9.76 ± 5.71(10^3^)	1.29 ± 1.87(10^3^)	*P* = 0.001
**S3**	2.00 ± 1.37(10^3^)	0.06 ± 0.24(10^3^)

**Table 7:** Pairwise comparison of the mean colony-forming units of Gram-positive and Gram-negative microorganisms between the groups

Groups	Gram-positive microorganisms	Gram-negative microorganisms	Wilcoxon test
**Group S1**	19.94 ± 9.87(10^3^)	6.47 ± 4.77(10^3^)	*P* = 0.001
**S2**	8.00 ± 6.20(10^3^)	2.35 ± 2.52(10^3^)	
**Group S1**	19.94 ± 9.87(10^3^)	6.47 ± 4.77(10^3^)	*P* = 0.001
**S3**	0.82 ± 1.33(10^3^)	1.00 ± 1.36(10^3^)	
**Group S2**	8.00 ± 6.20(10^3^)	2.35 ± 2.52(10^3^)	*P* = 0.01
**S3**	0.82 ± 1.33(10^3^)	1.00 ± 1.36(10^3^)	

## Discussion

The use of collected particulate bone as an autogenous bone graft during implant osteotomy eliminates the downsides of autogenous bone grafts such as increased duration of surgery, the need for a second surgical site, and donor site morbidity. However, contamination of collected bone with oral bacteria may increase the risk of peri-implant diseases and bone resorption [12]. Generally, during dental implant surgery, clinicians attempt to perform according to aseptic principles; however, previous studies revealed the risk of bacterial contamination of collected bone [3, 13].

Our study revealed the presence of various Gram-positive and Gram-negative bacteria in the collected bone when it was not treated with antibiotic. On the contrary, Blay et al. found no Gram-negative anaerobic microorganisms in collected bone [6]. Gram-negative anaerobic microorganisms are present in several forms of periodontal diseases and are known to produce enzymes capable of causing bone resorption [6].

Clindamycin has acceptable penetration into bone and is used for the treatment of bone infection and osteomyelitis [14, 15]. It is usually prescribed for treatment and prophylaxis of infection in oral and maxillofacial surgery [16]. Clindamycin is effective against *Staphylococcus aureus* and other Gram-positive cocci such as *Streptococcus pyogenes*, *Streptococcus pneumoniae*, and *Peptostreptococcus* species as well as Gram-negative anaerobic pathogens [10].

As *Staphylococcus* spp. can attach to the titanium surface to induce biofilm and cause peri-implantitis, it is important that increased exposure time to the intended antibiotic could overcome the strong cellular wall [10]. In addition, *Staphylococcus capitis* and *Staphylococcus epidermidis* are the most common pathogens of artificial valve endocarditis and deep tissue penetration with contaminated bone grafts could be harmful in susceptible patients.

Because of the high virulence of *Bacteroides* spp. in relation to skeletal tissue to induce small iatrogenic infections at surgical site and osseointegration disturbances, reduction of its population to zero in the least exposure time is important.

*Actinomyces* and *Peptostreptococcus* spp. are the etiologic factors of suppurative oral and maxillofacial infections and periodontal disease [16]. *Fusobacterium* spp. is one of the peri-implantitis pathogens like *Bacteroides* spp. So, killing all the colonies of these microorganisms after 3 min of exposure to the intended concentration of clindamycin is valuable.

*Pseudomonas aeruginosa* has a strong defense mechanism and penetration resistance to the most antibiotic agents and can live in the most antimicrobial environments. So, because clindamycin is not effective in the treatment of pseudomonas infections, the result of this study was predictable.

At low concentrations, clindamycin stimulates cell metabolism of human osteoblasts while it has cytotoxic properties at doses higher than 500 μg/mL. High doses of clindamycin can have adverse effects on human osteoblasts and may lead to potential alteration of bone metabolism in vivo [10]. Antibiotic-impregnated bone grafts have been successfully used in mandibular third molar sockets without impairing osteogenesis [17]. Witso et al. revealed that autogenous particulate bone soaked in antibiotics and grafted can deliver the drug to the grafting site [18].

Tezulas et al. introduced 3 min local application of chlorhexidine and clindamycin for bone graft decontamination. The concentration of their clindamycin solution was 150 mg/mL [12].

In the current study, local clindamycin at much lower concentration—20 μg/mL—was used for decontamination of collected bone graft and resulted in complete decontamination of four out of 17 samples. The outcomes supported the efficacy of very dilute local clindamycin (20 μg/mL) to reduce bacterial population. With an increase in antibiotic-bone graft contact time, a stronger antimicrobial effect can be expected. Cell viability after exposure to high concentrations of antibiotic solutions is an important factor for bone regeneration at the graft site. Sivolella et al. used rifamycin SV to reduce bacterial contamination of collected bone debris. They concluded that rifamycin SV (0.5% solution) could reduce bacterial contamination in collected material [19]. Etcheson et al. used tetracycline (50 mg/mL) for the decontamination of bone grafts. They demonstrated that tetracycline treatment caused a 7-fold decrease in streptococci count [3].

Clindamycin is not cytotoxic at low concentrations. Naal et al. demonstrated a significant increase in ALP-activity and increased matrix calcification due to the administration of 10 μg/mL concentration of clindamycin. Low concentrations of clindamycin increased cell metabolism of human osteoblasts without significant changes in cell number [10]. Clindamycin levels of 100–500 μg/mL may alter the metabolism and viability of osteoblastic cells in vivo [10].

In the investigation by Duewelhenke et al., 20% concentration for the proliferation of primary human osteoblast after 48 h incubation was determined as 20 μg/mL for clindamycin. In addition, the measurement of extracellular lactate concentration as an indicator of glycolysis revealed that high concentrations of clindamycin impaired mitochondrial energetics [11].

Antoci et al. revealed that ciprofloxacin at a dose greater than 100 μg/mL and vancomycin and tobramycin at doses greater than 2000 μg/mL significantly decreased cell proliferation. Therefore, the balance between the desired bactericidal properties and host cell toxicity is important for skeletal cell survival and function [20].

Clinical and laboratory studies showed that using a bacterial swab technique is insufficient for accurate assessment of decontamination techniques, and bone samples should undergo grinding before quantitative and qualitative microbial evaluations [21]. This method leads to technical bias. So, one of the advantages of this study is performing precise microbial evaluation along with bypassing the suggested grinding stage due to the nature of collected bone samples.

Pairwise comparison of total microorganism’s CFU between three study groups revealed statistically significant population reduction, in any fashion. So, although the selected clindamycin solution concentration is harmless for bone cells but selecting any of the mentioned decontamination methods—exposure time—could be according to the surgeon’s clinical judgment.

Some cross-sectional studies with wide heterogenicity report more implant failure rate following prescribing clindamycin as the prophylactic antibiotic in penicillin-allergic patients, compared with 2 gr amoxicillin in non-allergic patients [22, 23]. It seems that, not only more better designed trials in the form of randomized controlled clinical trial should be undertaken to analyze implant failure rate in penicillin-allergic patients with and without clindamycin, but also in vivo studies are needed to evaluate possible disruption in the bone healing and osseointegration process in these patients.

The result of this article is useful for the decontamination of other types of bone grafts.

This was an in vitro study to evaluate the bacterial population of particulate bone grafts after treatment with clindamycin (20 μg/mL). However, it did not focus on the viability of bone cells after exposure to antibiotic. This was a major limitation of this study. Further studies are encouraged to assess cell viability after the local use of clindamycin in different concentrations and exposure time.

## Conclusion

Local administration of clindamycin (20 μg/mL) is effective for the decontamination of particulate bone graft.

## Data Availability

The datasets used and/or analyzed during the current study are available from the corresponding author on reasonable request.
